# Progress in Medicine

**Published:** 1899-07-01

**Authors:** 


### Progress in Medicine.

### DISEASES OF THE LIVER AND PANCREAS.

Ch. Dubois[1] discusses the course by which bile is reabsorbed when it fails to pass away. His experiments are opposed to the view that the lymphatics are the chief agents in this process, which is the common theory. He finds that the greater part enters at once into the blood vessels, though a part does undoubtedly gain access to the lymphatics. A case of infective cholangitis caused by gall-stone in the common duct is reported by W. Hale White.[2] The patient, a woman of 30, had several attacks of pain, with jaundice and vomiting, followed by enlargement of the liver and high temperature, which lasted with intermissions for three months. Several gall-stones were passed during this period. Such pyrexia of severe and prolonged type may be produced by gall-stones, causing (a) ulceration of the common duct and fatal pyæmic abscesses in the liver, or (b) similar symptoms lasting many days, without abscesses and without a fatal result. This difference may perhaps be due to the difference in virulence of the streptococci or bacilli coli present. The diagnosis may be most difficult, but the patient in this case was for ten days free from pyrexia, and later on her rapid recovery showed that the disease had never gone on to the formation of pyæmic abscesses. Such cases of simple cholangitis are not common, but they are well worth noticing. The writer decided against operative interference in this case, as the patient's general health kept up, severe as the illness was; and the operation, when the liver is so enlarged, is a very serious one, with a mortality of probably 50 per cent.; besides, other stones may be formed later on. If there had been portal pyæmia no operation would have been of any use.

The microbic origin of calculi has been discussed by various authors. Gilbert[3] and others examined 70 calculi, and found the bacillus coli in one third of the number. They consider it proved that the microbe was present in the bile before the formation of the stone. They have also produced calculi by the injection of these bacilli into animals, and it would seem that the formation is a protective mechanism used for getting rid of a baneful intruder. Mignot has been able to produce calculi with regularity, and[4] lays down that the bacilli must be of weak virulence, and that in some way they must be prevented from premature expulsion from the gall bladder. The living microbes can be found afterwards in the middle of the calculus which is formed about them.

In the treatment of biliary obstruction E. F. Willoughby[5] records the successful use of Toluylene-diamine. The patient had suffered for two years every two or three days from biliary colic and vomiting, and the view was taken that the obstruction was due to thickened bile rather than to calculi. After various means had been tried, pills containing one grain of the drug were given once, and after awhile twice a day, with complete disappearance of the symptoms. The writer was led to make a trial of it from finding that it has the power of increasing both the solid and liquid constituents of the bile. The rapid and remarkable cure of the attacks of colic and of the jaundice under its use justify further trials of this remedy. T. E. Moore[6] remarks on the frequent troubles arising from the gall bladder, and compares it with the appendix both in this respect and in the comparative unimportance of its functions. He regards it as merely a safety-valve which acts only during the active flow of the bile, while many animals have none at all, and it has often been removed from human beings without injury. Inflammation of this organ generally arises from infection through the blood, and is accompanied with rigors, pyrexia, pain and a tender swelling in the right hypochondriac region. The diagnosis must be made from appendicitis, obstruction, typhoid, and pyelitis; but we have to remember the frequency with which cholecystitis complicates typhoid, and, indeed, pneumonia and other infective diseases. When distended, the bladder is prone to take infection, and if this distension arises from impaction of a gall stone in the common duct, we may find attacks of colic in addition to the other symptoms. Mild cases are best treated by rest, and a gentle laxative (calomel he distrusts); but severe cases go on to rupture, sepsis, or gangrene. To avoid this he recommends in them early incision and drainage.

**Abscess of the Liver.**—E. E. Field[7] holds that malaria, syphilis, and tuberculosis do not act directly as causes of abscess, but the injured tissues which they leave behind afford a suitable medium for the growth of pyogenic organisms in the circulation. Of direct causes dysentery is the most frequent, and he believes it is more often the cause than even Manson's figures indicate. A very common mistake is to regard the hectic of liver abscess as merely a symptom of malaria, though it is unaffected by quinine in full doses, and the spleen is but slightly enlarged. To locate the abscess a large needle should be introduced under chloroform with strict precautions, care being taken not to push down the piston during its withdrawal lest pus be driven into the peritoneum or pleura. The abscess when discovered should be freely drained. W. J. Buchanan[8] argues against the causation of abscess by dysentery. Dysentery, he says, is not a single disease, nor is the tropical form synonymous with the amœbic type, and it is exceedingly common, while abscess is rare. This rareness is still more marked in natives; and when outbreaks of dysentery occur, as in the Afghan War, there is no corresponding increase of abscesses. Thus, in 79,743 cases of dysentery in natives during 1892-6 there were only 127 of liver abscess. The dysentery which has been recorded in some cases has been only of a mild catarrhal form, and in others has occurred many years previously. In temperate climes abscess rarely occurs during outbreaks of dysentery. There was none in the Irish famine, only one case recorded in the Crimea, and very few, it is said, in the West Indies. Hence he would look for some other causes of abscess than this universally prevalent dysentery.

**Cirrhosis.**—Much interest has been caused by the proposal of R. F. Weir to cure intractable ascites due to cirrhosis by an operation which would form a collateral circulation through the omentum, and so re-establish the portal circulation. Janeway and Smith, in the discussion of his paper,[9] mentioned cases in which permanent relief had followed tapping, even when this had been repeated 65 times. Such instances Weir looks on as due to the natural formation of adhesions and of venous anastomoses. Vaughan Harley and Barratt,[10] in the course of a series of experiments to produce a true cirrhosis in animals, tried the effect of ligaturing the left hepatic duct while the other was left free, and found that there was an increase of the interlobular tissue, but without atrophy of the lobe, which accompanies true cirrhosis. They are now seeking to obtain more definite results by continuing the experiments for longer periods. Adami[1] found certain very minute organisms present in atrophic cirrhosis. Later on he discovered that these are a variety of the colon bacillus, and can be regularly found in healthy livers, but there they are always dead; whereas, in disease, they are often found alive. It is therefore possible that virulent bacilli may, when the liver activity is reduced by alcohol or otherwise, lead to cirrhosis, but further investigations are needed. The late Dr. Hanot[12] made a prolonged study of some cases of hypertrophic cirrhosis where he could exclude the agency of alcohol, as well as of malaria, syphilis, and tubercle. He noticed the constant existence of marked dyspepsia beforehand, and confirms Budd's view of its production by the absorption of the products of disordered digestion. Butyric, lactic, acetic, and other acids in this way may produce cirrhosis. Hasanlever[13] records three cases of hypertrophic cirrhosis, with chronic jaundice and large spleen, in three brothers and sisters. This, he thinks, was due to congenital syphilis, and makes out a strong case for this belief.

**Acute Yellow Atrophy.**—It is said that only about 200 cases in all of this affection have been reported. Dobie[14] discusses one in which recovery took place, and believes that the success was due to the regular and continued administration of small doses of oil of turpentine. It has a specific action on mucous membranes, and is both a tonic and stimulant, besides preventing hæmorrhage. In addition, he employed frequent doses of calomel, followed by saline aperients, with careful diet and fresh air.

**Pancreatic Carcinoma.**—Northrup and Hester[15] give a study of a case in a steady man, aged 46, who complained at first of pain in the back with dyspepsia. Great emaciation followed, while the gastric reactions were normal, and a somewhat fixed tumour in the middle line was at length discovered. Diarrhœa, with apparently fatty stools, continued to the end of his life. An analysis showed that 46·7 per cent. of the fat taken in his food could be collected from the fæces. In health there appears to be only about 10 per cent. thus lost. The excess may have been due to the absence of bile or of pancreatic juice, and previous observations are conflicting on the part played by the latter. The neutral fats which should be split up by pancreatic juice were not increased in the fæces, but this splitting up may have been carried out by the large number of micro-organisms in the intestines. Thus there was no means of deciding whether the state of the stools was due to the absence of bile or of pancreatic juice. No sugar was present. Mager[16] records a case of cancer of the body and tail of the pancreas with no jaundice, but a dark grey colouring of the face and neck. The fæces were partly grey and partly white, and contained much fat. Patches of fat necrosis were found around the pancreas. A case of necrosis of the pancreas[17] itself was operated upon by Morian for cholelithiasis. Septic symptoms followed, but the state of the pancreas and an abscess connected with it were only found post-mortem. Schlesinger[18] notices the rarity of lesions of the pancreas in acquired syphilis, but describes six cases of interstitial pancreatitis in the hereditary disease. The head is chiefly affected, and the organ feels hard and tough.

**Calculi of the Pancreas.**—Lancereaux[19] has collected forty cases, in twelve of which there was diabetes. Sedentary habits and overfeeding are predisposing causes, and we find chronic rheumatism and calculi of the liver or kidney are frequent concomitants. The stones may be numerous, or only one may be present. Suppuration or atrophy of the gland sometimes follows. Colic is not very frequent or severe, but is accompanied by temporary complete diabetes and fatty stools. In later stages permanent wasting diabetes may follow. The duration and prognosis are variable. The calculi may range in size from a pin's head to a hazel nut. Pearce Gould[20] records a case of pancreatic calculi, one of which obstructed the bile duct. Pain had been felt about the umbilicus, and jaundice came on. Some calculi were removed by operation, and after a severe attack of colic another was taken from the head of the pancreas. The biliary obstruction ceased, but the patient died from exhaustion. Kellock related a similar instance. Several cases of acute pancreatitis were described the same day at the Clinical Society. In one, reported by Messrs. Fripp and Bryant, intense pain above the umbilicus, with vomiting and collapse, ended fatally after exploration for supposed obstruction. Extensive hæmorrhage into the gastro-splenic omentum, the pancreas, and the surrounding tissues was discovered, together with much fat necrosis. A pure culture of the colon bacillus was found in the pancreas. In some of the cases a tumour was noticed above the umbilicus, which pulsated when the patient lay in certain positions, and in one or two instances the symptoms lasted for considerable periods. The more acute cases were thought to be due to embolism. Suppurative and gangrenous forms were held to be later stages of the hæmorrhagic type.

[1] Treatment, Jan. 12. [2] Lancet, Dec. 3. [3] Brit. Med. Jour., Jan. 14. [4] Brit. Med. Jour., Dec. 3. [5] Therapist, Mar. 15. [6] New York Med. Jour., Mar. 18. [7] New York Med. Jour., Feb. 25. [8] Jour. of Tropical Med., Feb. [9] Med. Rec., Jan. 28. [10] Brit. Med. Jour., Dec. 10. [11] Practitioner, Feb. [12] Brit. Med. Jour., Mar. 4. [13] Brit. Med. Jour., April 8. [14] Edin. Med. Jour., Dec. [15] Amer. Jour. Med. Sci., Feb. [16] Brit. Med. Jour., Jan. 21. [17] Loc. cit. [18] Brit. Med. Jour., Jan. 21. [19] Lancet, Mar. 11. [20] Lancet, Dec. 17.

